# Development and validation of interactive creativity task platform

**DOI:** 10.3389/fpsyg.2022.954946

**Published:** 2022-08-04

**Authors:** Ching-Lin Wu, Yu-Der Su, Eason Chen, Pei-Zhen Chen, Yu-Lin Chang, Hsueh-Chih Chen

**Affiliations:** ^1^Program of Learning Sciences, National Taiwan Normal University, Taipei, Taiwan; ^2^Institute for Research Excellence in Learning Sciences, National Taiwan Normal University, Taipei, Taiwan; ^3^Department of Educational Psychology and Counseling, National Taiwan Normal University, Taipei, Taiwan; ^4^Chinese Language and Technology Center, National Taiwan Normal University, Taipei, Taiwan

**Keywords:** creativity, divergent thinking, insight, dual process, computer scoring

## Abstract

Co-creativity focuses on how individuals produce innovative ideas together. As few studies have explored co-creativity using standardized tests, it is difficult to effectively assess the individual’s creativity performance within a group. Therefore, this study aims to develop a platform that allows two individuals to answer creativity tests simultaneously. This platform includes two divergent thinking tasks, the Straw Alternative Uses Test and Bottle Alternative Uses Test, and Chinese Radical Remote Associates Test A and B, which were used to evaluate their open-and closed-ended creative problem-solving performance. This platform has two modes: single-player mode and paired-player mode. Responses from 497 adults were collected, based on which the fluency, flexibility, and originality of divergent thinking were measured. This study also developed a computer scoring technique that can automatically calculate the scores on these creativity tests. The results showed that divergent thinking scores from computer-based calculation and manual scoring were highly positively correlated, suggesting that the scores on a divergent thinking task can be calculated through a system that avoids time-consuming, uneconomical manual scoring. Overall, the two types of tests on this platform showed considerable internal consistency reliability and criterion-related validity. This advanced application facilitates the collection of empirical evidence about co-creativity.

## Introduction

Creativity is a complex concept that involves different aspects that has been discussed at different levels (Sternberg et al., 2005; Benedek and Fink, 2019; Wu, 2019). In addition to the individual-level creative cognition mechanism (Wu et al., 2020b), the group-level co-creation process attracts growing attention (Zeilig et al., 2018). However, few studies have explored individuals’ creativity performance in interactive situations. Thus, it is a question of whether individuals can come up with a greater number of original ideas in a co-creation process. If this question is explored, the results of creativity research at the individual and group levels can be used to understand how cooperation affects individual creation.

Co-creativity or group creativity (Walsh et al., 2017), features that group members share knowledge, ideas, and their achievements, emphasizing inclusiveness, reciprocity, and relationship quality within a group. Co-creativity has been studied *via* drama creation (Zeilig et al., 2018) and story series (Schmoelz, 2018). However, standardized tests have seldom been used for co-creativity research. Thus, it is challenging to objectively and economically analyze individual creative performance and processes in groups or interactive situations. Therefore, this study aimed to develop an interactive creativity task platform that will provide a standardized tool for co-creativity research. Further, a platform will provide a more economical and objective form of assessment, thereby enhancing the understanding of individual creative problem-solving in interactive situations.

## Literature review

Among standardized creativity measures, divergent thinking and insight problem-solving are typical indices (Lin and Lien, 2013). According to dual process theory, they are open-and closed-ended problem-solving, respectively (Sternberg et al., 2005; Lin et al., 2012), which involve different intrinsic mechanisms (Lin and Lien, 2013). In brief, divergent thinking refers to individuals’ ability to generate various concepts *via* free association based on different semantic memory structures. This leads to creating unique thoughts (Guilford, 1956; Kenett and Faust, 2019), such as fluency (i.e., the number of responses), flexibility (i.e., the heterogeneity of a response), and originality (i.e., the appropriateness and uniqueness of a response). Additionally, insight problem-solving refers to the situation in which individuals change problem representations to overcome the impasses they encounter when solving problems, thus finding the right answers accompanied by an “aha” experience (Fleck and Weisberg, 2013; Weisberg, 2015).

The two creativity tests are described below. First, divergent thinking is measured based on structure-of-intellect theory, which includes four aspects: fluency, flexibility, originality, and elaboration (Guilford, 1956). Torrance (1974) developed the Torrance Tests of Creative Thinking (TTCT), which consisted of verbal and visual–spatial creative thinking tasks. In the TTCT, product improvement, unusual uses, asking questions, and circle subscales were used to evaluate respondents’ fluency, flexibility, originality, and elaboration. In Taiwan, Wu et al. (1998) developed the Chinese Version of the Creative Thinking Test (CVCTT), which also includes the above two parts: the verbal test requires respondents to think of unusual uses of bamboo chopsticks while the visual–spatial one asks respondents to draw a picture based on the pattern of the Chinese character “人” (jen; people). The former can be used to obtain fluency, flexibility, and originality, while the latter can be employed to collect the scores of fluency, flexibility, originality, and elaboration. Subsequently, Hsu et al. (2012) developed the Newspaper Alternative Uses Test (N-AUT), which can also be used to obtain the scores of fluency, flexibility, and originality. Good criterion-related validity existed between the N-AUT and the Chopstick Alternative Uses Test (C-AUT). It can be seen that the association of alternative uses is a widely used divergent thinking task because of its stable effects.

Moreover, insight problem-solving is an open-ended question with a closed-ended answer (Wakefield, 1992). Furthermore, the Remote Associates Test (RAT) shares a similar problem-solving process with insight problem-solving (Bowden and Jung-Beeman, 2003a). When individuals answer both tests, they will be misled into wrong problem representations and find it difficult to find the solution, whereas both will experience an “aha!” experience when solving a problem. The majority of empirical studies indicate that an individual’s RAT performance has a highly positive correlation with insight problem-solving (Huang et al., 2012; Chang et al., 2016). Therefore, RAT has been used to explore insight problem-solving (Bowden and Jung-Beeman, 2003b; Wu et al., 2016, 2020b). RAT was compiled by Mednick (1968) based on the associative hierarchy (Mednick, 1962): researchers select three remotely associated stimuli from commonly used verbal norms, asking respondents to find a stimulus that can be connected to all three stimuli. For instance, the three stimuli “blood, music, and cheese” make a question, and one possible answer to this question is blue, for the word “blue” can connect with the three stimuli to create the expressions “blue blood,” “blue music,” and “blue cheese,” respectively. The RAT boasts of a short duration of implementation, objective scoring, and simple compilation of test questions, which is conducive to the mass production of RAT questions to avoid question exposure before the test, therefore, it has been widely used in creativity research (Wu and Chen, 2017; Wu et al., 2020a). Moreover, it is believed to be an important tool that can be used to explore the process of creative generation in cognitive neuroscience (Wu et al., 2016, 2020b).

Remote Associates Test in Taiwan was compiled to the Chinese Remote Associates Test (CRAT) based on the pairing of Chinese characters (Jen et al., 2004). For instance, a CRAT question consists of three Chinese characters, i.e., “今” (chin; now), “輕” (ching; light), and “去” (chu; go), and one possible answer is the Chinese character “年” (nien; year), for it can pair with the three stimuli to form three meaningful Chinese words, i.e., “今年” (chin-nien; this year), “年輕” (nien-ching; being young), and “去年” (chu-nien; last year), respectively. They were the first to use a Chinese RAT to measure the remote association of a native Chinese speaker. Later, Wu et al. (2017) modified the way the CRAT was compiled to improve its validity and renamed it the Chinese Compound Remote Associates Test (CCRAT). Thereafter, Huang et al. (2012) and Chang et al. (2016) compiled CRATs based on the pairing of Chinese radicals and Chinese words. Three CRATs at all levels of Chinese vocabulary were compiled: The Chinese Radical Remote Associates Test (CRRAT; Chang et al., 2016), Chinese Word Remote Associates Test (CWRAT; Huang et al., 2012), and Chinese Compound Remote Associates Test (CCRAT; Wu et al., 2017). Comparatively, it is not easy to compile typical insight problems, for which test questions are susceptible to exposure before the test, so the present study used a remote associates test to measure one’s insight problem-solving ability. Among the three CRATs, the CRRAT has good criterion-related validity (Chang et al., 2016), so it is appropriate for the measurement of one’s insight problem-solving ability.

In addition, many studies have developed automatic scoring methods for creativity tests, including the alternate uses task (Beaty and Johnson, 2021), divergent thinking (Dumas et al., 2020), and creative writing (Zedelius et al., 2019). These studies have reported positive correlations between automatic scoring and manual scoring, indicating that automatic scoring is applicable to computerized creativity tests. Therefore, applying automatic scoring to an interactive creativity task platform will increase the convenience and efficiency of scoring and provide more instant feedback to respondents.

In summary, divergent thinking and insight problem-solving abilities are important indicators that measure creativity performance, representing different aspects of creativity. Consequently, they are suitable for measuring creativity in a group. Contrastingly, typical standardized creativity tests are pencil-and-paper tests, which make it difficult for multiple individuals to respond to the same test question simultaneously and refer to others’ answers. Additionally, the scoring of the divergent thinking tasks is often interpreted based on normative data to understand the type and originality of a response. Therefore, this study digitizes standardized creativity testing with a newly emerging technology—creating an online testing platform that simultaneously facilitates respondents to answer the questions. Moreover, natural language technology can be integrated into the platform to conduct scoring *via* a computer system. Thus, it provides researchers with an instant and objective way of scoring.

This study aimed to develop an interactive creativity task platform that allows two respondents to answer simultaneously to achieve the research purposes above. This platform includes two creativity tests: the divergent thinking tests [i.e., S-AUT and Bottle Alternative Uses Test (B-AUT)] and CRRAT (CRRAT A and B), with which it is hoped to evaluate one’s performance on open-and closed-ended creative problem-solving tasks. This platform allows two participants to respond to the same test question independently and with concerted effort. When two respondents perform a creativity task in a concerted effort, the platform collects data on their performance. Further, it collects data on whether they are inspired by others’ ideas, thus producing more original ideas or feeling easier to solve remote associative questions. This study collected the responses of divergent thinking test participants as normative data. Moreover, the reliability and validity of the two tests were analyzed. Therefore, participants’ creativity performance when they perform the task on their own and mutually can be compared, which can provide a reference for subsequent research.

## Materials and methods

### Participants

A total of 497 adults participated in this study voluntarily, of which 154 were men and 343 were women. They were aged between 20 and 30 years, with an average age of 23.50 (*SD* = 2.79). All participants were native Mandarin speakers with normal eyesight after the correction. This study was approved by the National Taiwan Normal University’s Institutional Review Board (IRB). All participants took part in the research only after they had understood it and signed an informed consent form. All participants were rewarded with the NTD$300 on completion of the task.

### Measures

This study developed an interactive creativity task platform that allows two persons to complete a creativity task simultaneously, while the platform consisted of two kinds of creativity tests: AUT and CRRAT. These two tests included two versions each. In addition, this study used typical creativity tasks as criterion tasks, including the divergent thinking test (Wu et al., 1998), CCRAT (Wu and Chen, 2017), CWRAT (Huang et al., 2012), and insight problem-solving (Chiu, 2005). The tools are described as follows.

#### Alternative uses test

This study compiled two divergent thinking tests (S-AUT and B-AUT) by referring to the existing AUT tests, such as the unusual use of bamboo chopsticks and newspapers (Wu et al., 1998; Hsu et al., 2012). In addition, this study gathered samples as normative data to calculate the scores of fluency, flexibility, and originality. To improve the accuracy of automatic scoring, the respondents were only allowed to answer the questions within five Chinese characters.

#### Chinese radical remote associates test

A total of 40 CRRAT questions were selected from the item pool compiled by Chang et al. (2016). The questions were divided into two parts, with an even number of test questions based on the degree of difficulty. Each CRRAT question is composed of three Chinese radicals, e.g., “女” (nü; female), “子” (tzu; son), and “禾” (ho; standing grain). Participants were required to propose a Chinese radical that can be paired with these three Chinese cues to create meaningful and commonly used Chinese characters. For this example, “乃” (nai; be) is one solution. The CRRAT participants were given one point for each correct answer. The higher the score, the better the remote associative ability.

The Cronbach’s α coefficient for the CRRAT compiled by Chang et al. (2016) was 0.70. It had a positive correlation with insight problem-solving (*r* = 0.42) but had no significant correlation with the indicators of the New Creativity Test (*r* fell between −0.10 and 0.15). Therefore, the CRRAT has good reliability, convergent validity, and discriminant validity.

#### Divergent thinking test

Compiled by Wu et al. (1998), the divergent thinking test includes verbal and visual–spatial data. This study only adopted the verbal one, which requires participants to think of unusual or creative uses of bamboo chopsticks that are often used to pick up food, so as to obtain fluency, flexibility, and originality scores. Fluency is the number of valid responses, flexibility is the number of categories of valid responses, and originality is the degree to which the response is different from the norm. The occurrence rate of response is higher than 5% (0 points), ranges from 2 to 5% (1 point), and less than 2% (2 points). Fluency was the sum of the scores for all valid responses.

In terms of scorer reliability, three dimensions (fluency, flexibility, and originality) of 20 tests were evaluated by different raters with expertise in the creative field. Further, the corresponding data on performance were used to calculate the Kendall harmony coefficient as the indices of scorer reliability, which included fluency (*r* = 0.96), flexibility (*r* = 0.97), and originality (*r* = 0.94).

#### Chinese compound remote associates test

In 2017, Wu and Chen compiled the CCRAT that was used in this study from which 20 test questions with different degrees of difficulty were selected. A CCRAT test question is composed of three stimuli Chinese characters, e.g., “療” (liao; treatment), “防” (fang; defense), and “統” (tung; completely). Participants were asked to think of a Chinese character that can pair with all three stimuli to create three meaningful two-character Chinese words. For this question, one solution is the Chinese character “治,” for it can be combined with the stimuli to form the two-character Chinese words “治療” (chih-liao; treatment), “防治” (fang-chih; prevention), and “統治” (tung-chih; ruling), respectively. Participants were given one point for each correct answer. The higher the score, the better the remote associative ability. For internal consistency reliability, the value of Cronbach’s *α* coefficient is 0.69, which is acceptable (Hung and Wu, 2021).

#### Chinese word remote associates test

Compiled by Huang et al. (2012), the CWRAT used in this study is also comprised of 30 questions. Each of its questions consists of three cues, e.g., “牛頓” (niu-tun; Newton), “蠟” (la; wax), and “紅色” (hung-se; red). Participants were asked to put forward a target Chinese word related to all of them. For this example, “蘋果” (ping-kuo; apple) is one possible answer. The participants were given one point for each correct answer. The higher the score, the better the remote associative ability. Their performance was represented by the pass rate (i.e., the percentage of correct answers).

The Cronbach’s α coefficient of the original scale is 0.81, and the criterion validity with respect to the insight problem shows a moderate positive correlation (*r* = 0. 51; Huang et al., 2012), showing that the CWRAT has acceptable reliability and validity.

#### Insight problem

Compiled by Chiu (2005), the Insight Problem is composed of six questions. The respondents were given one point for every correct answer and zero for each wrong answer. If respondents read a question before the test and knew its answer, the question was deemed invalid for them, so they were scored zero points even though they gave correct answers. Their performance is also represented by the pass rate


$$\left(i.e.,\frac{\mathrm{The number of correct answers}}{\mathrm{The number of valid questions.}}\right)$$

In terms of validity and reliability, Cronbach’s *α* coefficient was 0.52. Confirmatory factor analysis was used to analyze construct validity. The results were as follows: χ^2^(124) = 7.72, *p* > 0.05, GFI = 0.98, SRMR = 0.048, PNFI = 0.51, and CFI = 1.00; all indicators met the criteria of the overall fitness index, which means that the task was composed of a single latent factor. As for the fit of the internal structure, the loading of all factors reached a significant level, while the composite reliability reached 0.51, suggesting that the test met the criteria of the fitness index.

### Interactive creativity task platform

The interactive creativity task platform is a digitized system that provides online creativity tasks that record respondents’ AUT and CRRAT performance. It consists of two terminals: the user and administrator terminals.

**Figure 1:**
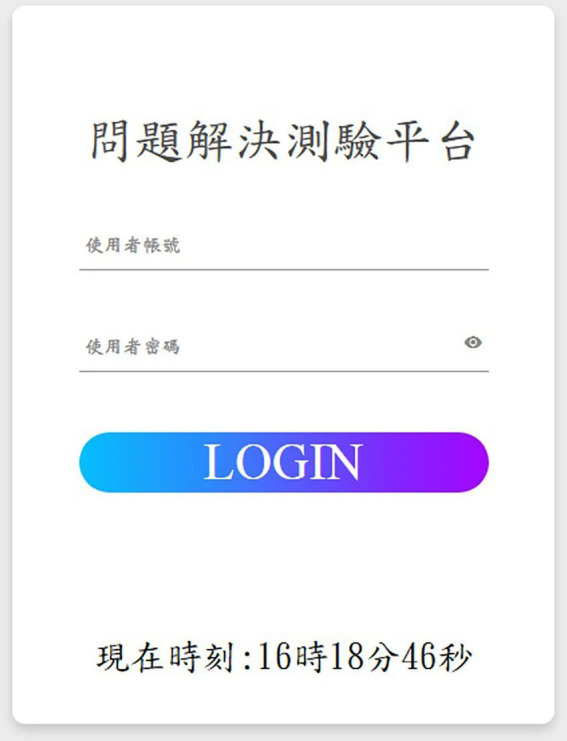
The login page of the interactive creativity task platform.

**Figure 2:**
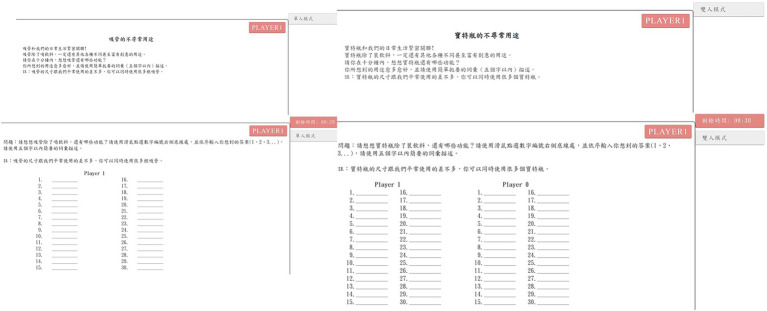
The user guide page and answering page of the interactive creativity task platform.

First, the user end has four webpages: login page, user guide page, answering page, and end page. The login page is the entry page of the platform; respondents are asked to key in given codes and passwords to log on to the platform, as shown in Figure 1. The user guide page offers instructions for creativity tasks. To avoid the situation in which respondents fail to read the instructions carefully, they are not able to pass this page unless the examiner allows it. The answering page consists of sections, such as question displaying sections, question-answering section, time remaining, and operating mode, as shown in Figure 2. In the paired-player mode, the respondents can see their counterparts’ responses, which they can use to form associations with more and different ideas. In particular, the respondents cannot talk to each other directly, but can only come up with more ideas by observing the responses of another respondent. The end page shows the message that the task has ended.

**Figure 3:**
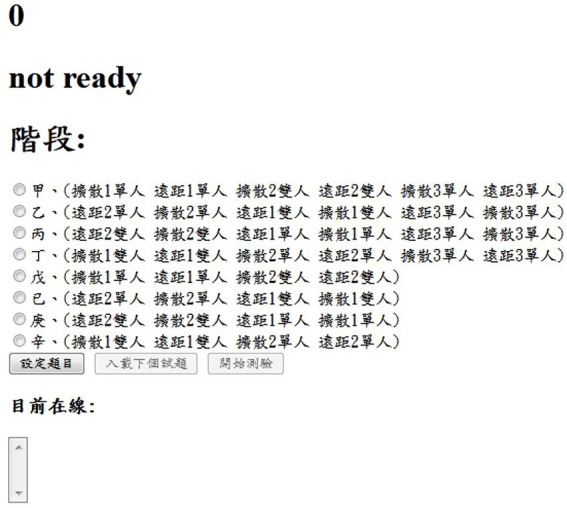
The administration page of the interactive creativity task platform.

Moreover, the administrator end has two types of webpages: a login page and an administration page. Examiners can only log on to the platform with accounts and passwords dedicated to an administrator. On the administration page, the examiner is authorized to decide the order in which the four creativity tests are carried out, as shown in Figure. 3. It is worth noting that only when examiners decide on the order of the tests can respondents log on to the platform to undertake them. In addition, respondents are all required to finish the four creativity tasks within 10 min, and examiners can set the number of respondents who are able to log on to the platform and undertake the same task at the same time. Finally, examiners export participants’ responses after the test is completed, including participants’ code, operating mode (single-or paired-player modes), the test that they have taken (S-AUT, B-AUT, CRRAT A, and CRRAT B), response code, response content, response time, etc.

### Procedure

This study was conducted in two groups. First, the experimental purpose and schedule were explained to the participants, after which they were asked to sign an informed consent form. The experiment consisted of two phases. Interactive creativity tasks were conducted on a computer during the first phase, including the S-AUT, B-AUT, CRRAT A, and CRRAT B. The participants completed the four tests in both the single-and paired-player modes. For instance, if respondents finished B-AUT in a single-player mode, they completed the S-AUT in a paired-player mode. Before they answered a test question in pairs, they would have a randomly chosen partner from those who were to finish the same test in the same time slot.

In the second phase, the respondents took part in the divergent thinking test and CCRAT, and 40 participants finished the CWRAT and Insight Problem. All tests in the first and second phase were performed following a counterbalanced design. The testing lasted approximately 80 min.

### Statistical analysis

As mentioned above, this platform includes S-AUT, B-AUT, CRRAT A, and CRRAT B. To understand the feasibility of these four tests, researchers collected participants’ responses to establish normative data for the AUT and examined their reliability and validity.

In terms of unusual uses, this study categorized participants’ respective responses to S-AUT and B-AUT by referring to the rules based on which normative data about unusual uses of bamboo chopsticks and newspapers were established (Wu et al., 1998; Hsu et al., 2012). In addition, researchers computed the number of a certain type of responses, and its creativity score was


$$\mathrm{set according to its frequency}\left(\frac{\mathrm{Number of}\;a\;\mathrm{type of response}}{\mathrm{Total number of responses.}}\right)$$

A response with a frequency above 5% had a creativity score of 0, that with a frequency between 2 and 5% had a creativity score of 1, and those with a frequency below 2% had a creativity score of 2. Therefore, the responses to the AUT were set as normative data, including the name, category, frequency, and creativity score of a response.

In addition, this study used Jieba (Chinese text segmentation),^1^ a system that can automatically break sentences into sense groups to distinguish participants’ responses from each other, to select major nouns of a response that were to be compared with normative data. In this way, the category and creativity scores of responses were obtained. The system then computed the AUT scores in terms of fluency, flexibility, and creativity.

The AUT performance of all participants was manually scored and calculated. A comparison between computer and manual scoring was performed to examine the consistency of the two scoring methods. Additionally, the correlation coefficients between S-AUT and C-AUT and between B-AUT and C-AUT were compared in terms of fluency, flexibility, and originality, based on which the criterion-related validity for the AUT was obtained.

As for CRRAT, participants’ responses were compared with standard answers based on whether they answered each question correctly. After that, the accuracy for the CRRAT participant was computed using the following equation:


$$\frac{\mathrm{The number of correct answers}}{\mathrm{Total questions.}}$$

In addition, we computed the internal consistency coefficient for each CRRAT test question. The correlation coefficient between the accuracy of CCRAT (both CRRAT A and B included) and that of CCRAT, CWRAT, and insight problem-solving, which was regarded as criterion-related validity.

Lastly, earlier studies have pointed out the reciprocity of co-creation (Walsh et al., 2017), such that individuals’ performance in the co-creation process is better than when working alone (Schmoelz, 2018; Zeilig et al., 2018). The researchers compared the differences in respondents’ performance on the four creativity tests in two different modes to understand whether the task mode influenced their creativity performance.

## Results and discussion

### Alternative uses test

Referring to the normative data on the AUT (Wu et al., 1998; Hsu et al., 2012), the responses to S-AUT and B-AUT were divided into 27 categories. The number of responses for each category is presented in Tables 1, 2. The responses were categorized into tools appear most frequently in the AUT, indicating that participants mainly regard bottles and straws as tools. Moreover, a number of the responses were categorized as stationery (*N* = 730, 744), cooking utensils (*N* = 553, 740), toys (*N* = 908, 726), and decorations (*N* = 786, 600), suggesting that the participants mainly used bottles and straws as the above items.

**Table 1:** Category and number of responses to B-AUT.

**Category**	**Times**	**Category**	**Times**	**Category**	**Times**
Tool	2,229	Transport	246	Teaching	62
Toy	908	Weapon	195	Sign	44
Decoration	786	Medical care	195	Currency	27
Stationery	730	Reproduction	184	Natural landscape	4
Exercise and fitness	623	Accessory	183	Body	4
Cooking utensil	553	Electrical equipment	182	Animal	3
Feeding	367	Furniture	154	Numerology	2
Music	347	Building	151	Celestial body	1
Science	345	Recreation	66	Symbol	1

**Table 2:** Category and number of responses to S-AUT.

**Category**	**Times**	**Category**	**Times**	**Category**	**Times**
Tool	1,994	Exercise and fitness	142	Furniture	54
Stationery	744	Teaching	128	Electrical equipment	39
Cooking utensil	740	Medical care	119	Animal	17
Toy	726	Feeding	98	Currency	15
Decoration	600	Building	82	Numerology	10
Accessory	322	Transport	72	Natural landscape	8
Science	300	Reproduction	64	Human body	6
Music	298	Sign	60	Symbol	5
Weapon	217	Recreation	56	Celestial body	2

Table 3 shows the means and SDs of the AUT scores from computer-based and manual scoring, as well as the correlation coefficients between the scores from the above two scoring methods. The results showed that the scores for each dimension obtained through computer-based and manual scoring were highly consistent (*r*s = 0.99, 0.92, 0.96, 0.99, 0.93, 0.95; *p*s < 0.001). These finding show that the automatic scoring used in the AUT yields results similar to manual scoring, and the effect sizes are greater than 80%. In particular, the high positive correlations in the two dimensions of flexibility and originality require human subjective classification, which is difficult. This result indicates that the automatic scoring of this platform has favorable credibility. In terms of the differences between the two scoring methods, the means of fluency and flexibility were less than 0.2, indicating that the system was able to effectively identify the types of responses. In addition, the gap between computer-based and manual scoring of originality fell between 0.1 and 1.3, which means that computer-based scoring may underestimate respondents’ score for fluency. This is probably because the system would compare major nouns of a response with normative data if the system found that the response was not completely the same as the normative data when a fuzzy comparison between a response and normative data was conducted. If the major nouns of a response belong to a category that has a high frequency, its creativity score will be low. However, it was a systematic error, so it probably exerted a limited impact on the within-group differences. If subsequent research uses computer-based and manual scoring at the same time, it would be feasible to add one point to the creativity score if it is calculated based on computer scoring to bridge the gap between computer-based and manual scoring.

**Table 3:** Differences in Computer and Manual Scoring for AUT.

	*N*	Computer scoring		Manual scoring		*r*	*t*	*p*	*d*
		*Mean*	*SD*	*Mean*	*SD*				
S-AUT-fluency	488	13.42	6.72	13.28	6.71	0.99	3.46	0.00	0.16
S-AUT-flexibility	488	6.97	2.49	7.06	2.63	0.92	−1.93	0.05	0.09
S-AUT-originality	488	13.56	8.92	14.63	10.14	0.96	−7.74	0.00	0.35
B-AUT-fluency	492	16.43	6.53	16.30	6.63	0.99	3.21	0.00	0.14
B-AUT-flexibility	492	8.64	2.60	8.72	2.74	0.93	−1.87	0.06	0.08
B-AUT-originality	492	14.20	8.47	14.27	9.15	0.95	−0.49	0.62	0.02

S-AUT, Straw Alternative Uses Test; B-AUT, Bottle Alternative Uses Test.

Table 4 lists the correlation coefficients of the AUTs in different dimensions, including S-AUT, B-AUT, and C-AUT. It shows that S-AUT and B-AUT have a positive correlation with the divergent thinking test (Wu et al., 1998) in terms of fluency, flexibility, and originality (*rs* = 0.79, 0.54, 0.58, 0.75, 0.51, 0.60, *ps* < 0.001), suggesting favorable criterion-related validity between S-AUT and B-AUT. In addition, Table 5 displays correlation coefficients between the AUTs (B-AUT and S-AUT) and CRRAT (CRRAT A and B). The test scores between AUT and CRRAT had a low positive correlation, indicating that the divergent thinking test and the CRRAT used on the interactive creativity task platform have appropriate discriminant validity.

**Table 4:** Correlation coefficients among the three AUTs.

	1	2	3	4	5	6
S-AUT-fluency	-					
S-AUT-flexibility	0.83^**^	-				
S-AUT-originality	0.91^**^	0.77^**^	-			
B-AUT-fluency	0.74^**^	0.62^**^	0.68^**^	-		
B-AUT-flexibility	0.61^**^	0.55^**^	0.55^**^	0.83^**^	-	
B-AUT-originality	0.70^**^	0.58^**^	0.70^**^	0.87^**^	0.68^**^	-
C-AUT-fluency	0.79^**^	0.65^**^	0.72^**^	0.75^**^	0.60^**^	0.69^**^
C-AUT-flexibility	0.63^**^	0.54^**^	0.58^**^	0.59^**^	0.51^**^	0.54^**^
C-AUT-originality	0.65^**^	0.52^**^	0.58^**^	0.63^**^	0.48^**^	0.60^**^

S-AUT, Straw Alternative Uses Test; B-AUT, Bottle Alternative Uses Test; and C-AUT, Chopsticks Alternative Uses Test. **p < 0.01.

**Table 5:** Correlation coefficients between AUTs and CRRATs.

	CRRAT A	CRRAT B
S-AUT-fluency	0.21^**^	0.18^**^
S-AUT-flexibility	0.15^**^	0.18^**^
S-AUT-originality	0.14^**^	0.10^*^
B-AUT-fluency	0.22^**^	0.14^**^
B-AUT-flexibility	0.20^**^	0.17^**^
B-AUT-originality	0.13^**^	0.05

S-AUT, Straw Alternative Uses Test; B-AUT, Bottle Alternative Uses Test; and CRRAT, Chinese Radical Remote Associates Test. *p < 0.05, **p < 0.01.

### Chinese radical remote associates test

The average pass rates of CRRAT A and B were 0.40 (*SD* = 0.19) and 0.39 (*SD* = 0.20). The internal consistency coefficients were.80 and.79, respectively, both falling within the acceptable range, which indicates that the CRRAT has stable internal reliability. Moreover, CRRAT was significantly and positively correlated with CCRAT (*rs* = 0.24, 0.24, *ps* < 0.001, *N* = 464), CWRAT (*rs* = 0.41, 0.48, *ps* < 0.01, *N* = 50), and insight problem-solving (*rs* = 0.44, 0.35, *ps* = 0.002, 0.012, *N* = 50), as shown in Table 6. The positive correlation indicates that CCRAT A and B have good criterion-related validity, which is consistent with the findings of previous research (Chang et al., 2016).

**Table 6:** Criterion-related validity of CRRAT scores.

	CRRAT A	CRRAT B	CCRAT	CWRAT	Insight
CRRAT A	0.80^**1^	0.51^**^	0.24^**^	0.41^**3^	0.44^**4^
CRRAT B	0.51^**^	0.79^**2^	0.24^**^	0.48^**3^	0.35^*4^

CRRAT, Chinese Radical Remote Associates Test; CCRAT, Chinese Compound Remote Associates Test; CWRAT, Chinese Word Remote Associates Test; Insight, insight problem-solving; 1 and 2 stand for internal consistency coefficients, 3 means that the *N* of the two tests is 52, while 4 indicates that *N* of the two tests is 50. *p < 0.05, **p < 0.01.

### The creativity performance in single-and paired-player modes

To understand whether paired-player interaction mode influences individual creativity, the present study examined the differences in creativity test performance under different task modes. In particular, the number of participants in the experiment is not always even, because some participants were suddenly absent. If the number of participants in this experiment is odd, the participant ordered last will perform all tasks in single-player mode. Therefore, the overall number of participants in the single-player mode is larger than in the paired-player mode. The respondents’ performance on the AUTs and CRRATs in single-and paired-player modes is shown in Table 7. First, the fluency score of S-AUT was significantly higher in the single-player mode than in the paired-player mode (*t* = 2.07, *p* = 0.039, *d* = 0.19), but the other dimensions showed no significant differences between the two modes (*t*s < 1.88, *p*s > 0.06, *d*s < 0.17). Nevertheless, the difference in fluency scores was lower than the effect size, indicating that respondents did not produce a greater number of more flexible and original ideas because they were able to refer to others’ ideas in the paired-player mode. In addition, the accuracy of CRRAT was higher in the paired-player mode than in the single-player mode (*t*s = −3.98, −3.01, *p*s < 0.005, *d*s = 0.36, 0.27), which indicates that one is more likely to come up with correct answers by referring to others. To summarize, this study reveals that the paired-player mode may exert different impacts on the divergent thinking test and RAT.

**Table 7:** Differences in AUT and CRRAT Scores under single-and paired-player modes.

	Single-player			Paired-player			*t*	*p*	*d*
	*N*	*Mean*	*SD*	*N*	*Mean*	*SD*			
S-AUT-fluency	277	13.86	6.59	211	12.60	6.80	2.07	0.039	0.19
S-AUT-flexibility	277	7.01	2.40	211	6.81	2.53	0.91	0.364	0.08
S-AUT-originality	277	14.01	9.31	211	12.46	9.45	1.81	0.071	0.16
B-AUT-fluency	276	15.90	6.43	216	16.61	6.53	−1.21	0.226	0.11
B-AUT-flexibility	276	8.71	2.60	216	8.61	2.47	0.43	0.668	0.04
B-AUT-originality	276	12.64	8.32	216	14.09	8.68	−1.88	0.060	0.17
CRRAT A	274	0.37	0.19	215	0.44	0.18	−3.98	0.000	0.36
CRRAT B	273	0.38	0.20	210	0.43	0.19	−3.01	0.003	0.27

S-AUT, Straw Alternative Uses Test; B-AUT, Bottle Alternative Uses Test; and CRRAT, Chinese Radical Remote Associates Test.

## General discussion

Co-creativity focuses on how individuals produce original ideas *via* teamwork, thus producing the effect of “1 + 1 > 2” (Woodman et al., 1993; Walsh et al., 2017). Unfortunately, few standardized tools measure co-creativity. Therefore, this study developed an interactive creativity task platform that allows two individuals to interact, through which one’s creativity in an interactive situation is analyzed. This platform uses AUT (S-AUT and B-AUT) and CRRAT (CRRAT A and B) to measure open-and closed-ended problem-solving abilities, respectively. This study collected responses to the above four tests from nearly 500 adults, and the corresponding data were used as the normative data of the two divergent thinking tests to calculate the scores of fluency, flexibility, and originality. Moreover, the differences between the two divergent thinking test scores from computer and manual scoring were analyzed, and the results showed appropriate inter-rater reliability, which validates the stability of computer scoring. In brief, the scores of participants’ responses to the divergent thinking test questions can be calculated *via* the platform, which improves the disadvantages of manual scoring, which is time-consuming and uneconomical. In addition, CRRAT A and CRRAT B displayed stable internal consistency. Subsequently, the correlations between the four tests and criterion-related validity tasks were analyzed. It was found that the four creativity tests used on the platform had good discriminant and convergent validity (Wu et al., 1998; Hsu et al., 2012; Huang et al., 2012; Chang et al., 2016).

In addition, a comparison of creativity performance in the single-and paired-player modes finds better performance in the paired-player mode during closed-ended creative problem-solving, whereas there tends to be similar performance in the two modes during open-ended creative problem-solving. This result suggests that paired interaction helps to solve creative problems with objective answers, but has limited benefit for creative problems with no standard answers. These findings corroborate past studies regarding the positive influence of co-creation on individual creativity (Schmoelz, 2018; Zeilig et al., 2018). Accordingly, the results show that paired interactions have different influences on different types of creative problem solving.

In general, this study has successfully established online tests with stable reliability and validity, which measures open-and closed-ended problem-solving abilities in single-and paired-player modes. The establishment of this platform pioneers the study of two participants responding to a creativity test simultaneously. In addition, computer-based scoring was developed for this platform, affording researchers access to the respondents’ scores during testing in real time. The results of this study echo the findings regarding the automated scoring of creativity tests in recent years (Zedelius et al., 2019; Dumas et al., 2020; Beaty and Johnson, 2021), and further show that automatic scoring technology yields similar scores to manual scoring. This computerized development will render the scoring of creativity tests more convenient and objective in future research.

These results have some empirical applications. Future co-creativity research can use the platform to meet specific needs. For instance, if researchers wish to explore creativity in cooperative or competitive situations, they can adopt computer-based scoring to obtain scores for each test in real time. In addition, researchers can use the platform to explore individuals’ intrinsic characteristics, such as motivation, character, and self-efficacy, as well as their creativity in single-and paired-player modes, thus facilitating the analysis of how such characteristics affect creativity in the two modes. However, the stimulus and scoring norms of the test platform are all in Chinese, which limits such research to Chinese-speaking populations. Nonetheless, these applications promise to enrich the empirical base of co-creativity research that can serve as reference for future studies.

## Data availability statement

The raw data supporting the conclusions of this article will be made available by the authors, without undue reservation.

## Ethics statement

The studies involving human participants were reviewed and approved by Institutional Review Board at National Taiwan Normal University. The patients/participants provided their written informed consent to participate in this study.

## Author contributions

C-LW contributed to data collection, data analysis, data interpretation, and writing. Y-DS and EC contributed to programming. P-ZC and Y-LC contributed to data analysis. H-CC supervised the project. All authors contributed to the article and approved the submitted version.

## Funding

This work was financially supported by the “Institute for Research Excellence in Learning Sciences” of National Taiwan Normal University (NTNU) from the Featured Areas Research Center Program within the framework of the Higher Education Sprout Project by the Ministry of Education (MOE) in Taiwan. We also thank the Ministry of Science and Technology, Taiwan, R.O.C. for funding this study, through projects on “How is one plus one more than two? Exploring the process and training of creative thinking and creative product under interactive situations: From measurement, mechanisms to neural plasticity” (MOST108-2410-H-003-080) and “How do individuals display their creativity in groups? The moderation effects of intrinsic and extrinsic factors, brain structure and interactive mobile teaching” (MOST109-2628-H-003-005-MY2).

## Conflict of interest

The authors declare that the research was conducted in the absence of any commercial or financial relationships that could be construed as a potential conflict of interest.

## Publisher’s note

All claims expressed in this article are solely those of the authors and do not necessarily represent those of their affiliated organizations, or those of the publisher, the editors and the reviewers. Any product that may be evaluated in this article, or claim that may be made by its manufacturer, is not guaranteed or endorsed by the publisher.

## Footnotes

^1^ https://github.com/fxsjy/jieba
